# Chondrocyte Aging: The Molecular Determinants and Therapeutic Opportunities

**DOI:** 10.3389/fcell.2021.625497

**Published:** 2021-07-14

**Authors:** Thamil Selvee Ramasamy, Yong Mei Yee, Ilyas M. Khan

**Affiliations:** ^1^Stem Cell Biology Laboratory, Department of Molecular Medicine, Faculty of Medicine, Universiti Malaya, Kuala Lumpur, Malaysia; ^2^Cell and Molecular Biology Laboratory, The Dean’s Office, Faculty of Medicine, Universiti Malaya, Kuala Lumpur, Malaysia; ^3^Centre of NanoHealth, Swansea University Medical School, Swansea, United Kingdom

**Keywords:** senescence, osteoarthritis, chondroprotection, degeneration, regenerative medicine

## Abstract

Osteoarthritis (OA) is a joint degenerative disease that is an exceedingly common problem associated with aging. Aging is the principal risk factor for OA, but damage-related physiopathology of articular chondrocytes probably drives the mechanisms of joint degeneration by a progressive decline in the homeostatic and regenerative capacity of cells. Cellular aging is the manifestation of a complex interplay of cellular and molecular pathways underpinned by transcriptional, translational, and epigenetic mechanisms and niche factors, and unraveling this complexity will improve our understanding of underlying molecular changes that affect the ability of the articular cartilage to maintain or regenerate itself. This insight is imperative for developing new cell and drug therapies for OA disease that will target the specific causes of age-related functional decline. This review explores the key age-related changes within articular chondrocytes and discusses the molecular mechanisms that are commonly perturbed as cartilage ages and degenerates. Current efforts and emerging potential therapies in treating OA that are being employed to halt or decelerate the aging processes are also discussed.

## Introduction

Articular cartilage is a highly specialized connective tissue found at the ends of the articulating bones that allows transmission of forces and provides a smooth surface for low-friction movement of weight-bearing joints. Articular cartilage is composed of a dense extracellular matrix (ECM) with a sparse distribution of highly specialized cells called chondrocytes. It is avascular, aneural, and alymphatic in nature; hence, it has a poor self-repair capacity, and therefore damage to cartilage in weight-bearing joints is at a higher risk of progressing into more serious joint conditions such as osteoarthritis (OA).

Aging is also a major risk factor for the development of OA. Age-related changes in articular cartilage predispose individuals to develop OA; additional factors including biomechanical, genetic, or systemic metabolic factors can accelerate the progression of the condition (Del Carlo and Loeser, 2003). It is generally accepted that OA is a multifactorial disease and, this supersedes a more simplistic notion of it being a disease of “wear and tear.” Newer mechanisms attributed to the disease include cell senescence and the senescence-related secretory cell phenotype, chondrocytes’ reduced reactivity to growth factors, mitochondrial dysfunction, oxidative stress, and abnormal accumulation of advanced glycation end products (AGEs). It is the cumulative effect of the mechanical load and associated cell dysfunction over the years that result in cartilage breakdown and the macroscopic clinical evidence of “wear and tear.” Hence, OA is regarded as a naturally occurring irreversible phenomenon, rather than a specific, potentially treatable disease (Loeser, 2011); however, in light of new studies, this paradigm is being challenged, and new thinking may lead to novel medical approaches for the prevention, reversal, or treatment of OA. Therefore, this mini-review highlights the molecular mechanisms underlying aging of chondrocytes and degeneration of cartilage.

## Molecular and Cellular Regulation

### Proteostasis (Autophagy, UPS, and Protein Folding Mechanism)

One hallmark of aging is loss of proteostasis caused by dysfunctional ubiquitin-proteasome system (UPS), protein folding, and autophagy. Age-related decreases in proteostatic activity impact cellular differentiation and viability and inflammatory processes in disease. Indeed, impaired proteasomal function in human osteoarthritic chondrocytes can contribute to decreased levels of sox9 and aggrecan, factors that are crucial for chondrocyte function and maintenance (Serrano et al., 2018). Immunoproteasomes reflects a persistent antistress mechanism in aging tissue. Activation of forkhead transcription factor (FoxO) in response to reduced IGF-1 signaling enhances longevity (Löw, 2011). These findings indicate that IGF-I or insulin can reduce protein degradation rapidly by suppressing autophagy *via* mTOR activation and independently Akt suppressing FoxO transcription, which also inhibits proteasomal degradation through the reduction of transcription of ubiquitin ligases atrogin-1 and MuRF1 (Vellai et al., 2009). It is noteworthy that age-related decline in expression of molecular chaperones induces endoplasmic reticulum (ER) stress and cellular apoptosis in articular cartilage (Tan et al., 2020), which suggests that loss of proteostasis induces ER stress in aged articular chondrocytes. Autophagy is one arm of the proteostasis network that coordinates proteome and organelle quality control and degradation as well as the regulation of energy and nutrient supply, thereby maintaining cell survival and normal biosynthetic function in virtually all cell types. Autophagy is necessary for lifespan extension in several organisms, and multiple autophagy-related proteins are directly modulated by longevity pathways. Autophagy is a protective and homeostatic mechanism in normal cartilage especially in modulating cellular responses to stress. If autophagic pathways are compromised, cells may undergo apoptosis, leading eventually to cartilage degeneration (Caramés et al., 2010). Autophagy-related protein-7 is an essential regulator of autophagosome assembly; when depleted in chondrocytes, they accumulate large numbers of glycogen granules, hardly proliferate, and died specifically in the proliferative zone without any ER-stress signal (Horigome et al., 2020). Suppression of autophagy in prechondrogenic cells leads to defective chondrogenesis, through a lack of glycogenolytic supply of glucose to avascular prechondrocytes. Enhanced autophagy is reported to affect intracellular metabolic activity, i.e., by regulating the metabolism of nutrients, protein, and lipid and can delay the progression of OA (Luo et al., 2019). Intriguingly, key bioenergy sensors such as the AMP-activated protein kinase (AMPK) signaling pathway and Sirtuin 1 (Sirt1) also have roles in the regulation of autophagy, senescence, and aging (Ong and Ramasamy, 2018).

Conversely, cellular regulators of autophagy such as Sirt1, forkhead family O subclass transcription factor 3 (FoxO3), mammalian target of rapamycin (mTOR), nuclear factor-κB (NF-κB), and p53 have pivotal roles in energy metabolism, gene and protein expression, and aging. Sirtuin, known for its roles in stress resistance and longevity, and FoxO3, a major modulator of cellular metabolism, proliferation, and oxidant stress resistance, enhance autophagy, whereas mTOR, and NF-κB repress autophagy following stress and inflammation, respectively (Lotz and Caramés, 2011). The loss of autophagy in articular cartilage under mechanical or inflammatory stress is associated with aging-related cell death and increasing OA severity (Goldring and Berenbaum, 2015). Experimental evidence suggests that autophagy plays both a cytoprotective and death-promoting role in the pathogenesis of OA (Chang et al., 2013). Autophagy is activated as an adaptive response to hypoxic conditions; it also plays a cytoprotective role under various types of stress including disease treatment with DNA-damaging reagents, ER stress, nutrient and energy deprivation, as well as radiation. Overall, the age-dependent decline in autophagic activity contributes to the accumulation of damaged macromolecules and susceptibility to aging-related OA (Caramés et al., 2010). In contrast to autophagy-induced cell survival, the occurrence of OA autophagy in OA chondrocytes may be over-induced to the extent that the essential cellular constituents for cell survival are degraded leading to autophagic cell death. The mechanisms by which autophagy regulates the pathogenesis of OA have yet to be fully unraveled (Chang et al., 2013); however, it seems understanding its role holds immense potential in targeting the process to modulate aging of chondrocytes and eventually may be a promising therapy for treating OA.

### Mitochondrial Dysfunction

Mitochondrial oxidative phosphorylation accounts for up to 25% of the total steady-state adenosine triphosphate (ATP) production in cartilage; however, in OA chondrocytes, mitochondrial functions including mitochondrial respiratory chain (MRC) activity and ATP synthesis are altered. Intriguingly, proteomic analysis of healthy and OA cartilage reveals 26% of the deregulated protein signature is related to respiratory chain function (Ruiz-Romero et al., 2009). The accumulation of mitochondrial DNA (mtDNA) deletions and point mutations or the indirect effects of nitric oxide (NO), proinflammatory cytokines, prostaglandins, and reactive oxygen species (ROS) on MRC function and ATP synthesis could lead to chondrocyte dysfunction. For example, deficiency of mitochondrial superoxide dismutase 2 and increases ROS in chondrocytes leads to mitochondrial dysfunction (Gavriilidis et al., 2013).

Thus when ROS generation exceeds the antioxidant activity threshold of chondrocytes, oxidative stress impairs MRC protein complexes resulting in reduced ATP production, deprivation of energy reserve, impaired matrix synthetic function, and reduced chondrocyte viability. Importantly, mitochondrial dysfunction affects several pathways that are critically involved in OA pathology, including oxidative stress generation, chondrocyte apoptosis, cytokine-induced chondrocyte inflammation, and matrix catabolism, as well as ECM calcification (Blanco et al., 2011). MRC dysfunction perturbs the homeostatic balance of healthy cartilage by inducing the production of proinflammatory stimuli and matrix metalloproteinases promoting catabolic glycosaminoglycan release, while simultaneously suppressing the synthesis of proteoglycans thereby exacerbating cartilage degeneration. Certain mtDNA haplogroups predispose people to OA; mtDNA haplogroup U is associated with an increase in radiologic severity of knee in OA, conversely mtDNA haplogroup J safeguards against hip and knee in OA (Blanco et al., 2011; Goldring and Berenbaum, 2015). AMPK and Sirt1 work together to maintain biological homeostasis through suppressing oxidative stress, NF-κB activation, and deregulation of several inflammatory and catabolic responses. NF-κB activation of inflammatory and catabolic responses is suppressed by deacetylating the p65 NF-κB subunit and priming it for proteasomal degradation, resulting in enhancing autophagy *via* repair of dysfunctional mitochondria. However, deficiency of AMPK activation in OA and aging chondrocytes could lead to reduced expression of Sirt1 and peroxisome proliferator-activated receptor g coactivator 1α (PGC-1α), thereby contributing to chondrocyte mitochondrial dysfunction. New evidence has demonstrated that pharmacologic activation of AMPK inhibits inflammation-induced catabolic activities, upregulates expression of antioxidant enzymes and prevents excessive mitochondrial ROS production. Importantly, activation of the AMPK/Sirt1/PGC-1α signaling pathway reverses impaired mitochondrial biogenesis capacity in human OA chondrocytes via mitochondrial transcription factor A (TFAM) mediation. The concept of therapeutic activation of chosen components of the AMPK/Sirt1/PGC-1α pathway is yet to be validated in an *in vivo* animal model of OA and in human OA (Wang et al., 2015). In addition to Sirt1, a recent study has highlighted the role of Sirt3-mediated mitochondrial homeostasis in OA. Sirt3, which is mainly located in mitochondria, can exert its deacetylation activity to regulate mitochondrial function, regeneration, and dynamics (He et al., 2020). Mitochondrial dysfunction-induced chondrocyte phenotypic inflammatory and matrix degradation responses also occur *via* ROS-mediated activation of c-Jun N-terminal kinase (JNK)-mitogen-activated protein kinase (MAPK)/cFos-AP1 pathways in chondrocytes of osteoarthritic and aged cartilage (Ansari et al., 2020). Although ROS generation in cells is inevitable, in human chondrocytes autophagy activation protects against mitochondrial dysfunction caused by accumulated ROS damage. Taken together, an intimate and highly coordinated link between bioenergy systems and chondrocyte aging or OA is now evident. This link is regulated through a balanced redox system, protective mechanisms such as autophagy, and apoptosis-survival/longevity pathways such as JNK-MAPK/cFos-AP1 and AMPK/Sirtuins pathways (López de Figueroa et al., 2015).

### Oxidative Stress

Oxidative stress ensuing from an imbalance of ROS synthesis and antioxidant defense is a result of increased ROS synthesis or decreased level of antioxidant and can be measured in chondrocytes by, for example, an increased level of nitrotyrosine, a measure of ROS-induced oxidative damage to proteins (Loeser et al., 2002; Del Carlo and Loeser, 2003; Hui et al., 2016), and an increased ratio of oxidized glutathione to reduced glutathione with age (Del Carlo and Loeser, 2003). *In vitro* studies suggest that when under oxidative stress cellular antioxidant enzymes are inactivated *via* nitration of catalytically active tyrosine residues; Tyr106 and Tyr104 (Savvides et al., 2001). Furthermore, peroxynitrite (ONOO-), a potent oxidant formed from the reaction of NO with superoxide, is probably responsible for the inactivation of thiol-related antioxidant enzymes (Del Carlo and Loeser, 2003). With an imbalanced redox status, the susceptibility of chondrocytes to oxidant-mediated cell death increases, and albeit indirectly, predisposes the older individuals to develop OA (Del Carlo and Loeser, 2003). In response to inflammatory mediators, mechanical stress, and partial oxygen pressure (pO_2_), chondrocytes can produce an abnormal level of ROS that exceed their antioxidant capability leading to a disturbance of redox homeostasis. Overproduction of ROS oxidizes membrane phospholipids, intracellular and extracellular components, nucleic acids, and transcription factors, leading to impaired biological activity and cell death. ROS or secondary byproducts of oxidative stress likely lead to oxidation of collagens and proteoglycans by covalently modifying the primary structure of the proteins. ROS induces oxidative cleavage of collagens and proteoglycan by breaking the amino acid bonds or amino acid side chains. Additionally, oxidative posttranslational modifications induce the unfolding of collagens and proteoglycans employing steric hindrance or by altering hydrogen bonds and electrostatic interactions. Ultimately, crosslinking within the proteins or between neighboring proteins give rise to alterations in protein secondary and tertiary structure, the spatial orientation of collagen fibers and bundles, as well as surface charge and tension of proteins, which all impair the biomechanical properties of ECM. Initially, oxidative stress causes posttranslational modification of ECM proteins, and following the second set of signals, including AGE- and ROS-induced inflammation and catabolic pathways, act in conjunction to promote degeneration of articular cartilage, leading to the OA phenotype (Hardin et al., 2015). Stress-induced chondrocyte apoptosis is mediated *via* PI3K/Akt and caspase pathways at the very early stages of cellular stress (Lee et al., 2020). Indeed, extensive oxidative stress decreases the synthesis of collagen and proteoglycan *via* regulation of phosphatase and tensin homolog deleted on chromosome 10 (PTEN), which negatively regulates PI3K/Akt and ERK/MAPK pathways endogenously, and these pathways are essential for the synthesis of ECM proteins. Interestingly, a recent study has highlighted that the prolonged activation of Akt signaling caused an accumulation of ROS and triggered chondrocyte senescence as well as senescence-associated secretory phenotype (SASP) in PTEN-deficient mice (Xie et al., 2019). On the other hand, chronic administration of the antioxidant *N*-acetylcysteine suppresses chondrocyte senescence (Xie et al., 2019), suggesting the vital role of an antioxidant in mitigating aging of chondrocytes and OA progression. Collectively, degradation products and cellular content embodying oxidized molecules could aggravate synovial inflammation and create a vicious cycle, established by further degradation of products and newly synthesized ROS (Henrotin et al., 2003). Newly discovered evidence shows that a progressive loss of cartilage ECM and cellularity with advancing age is associated with elevated levels of oxidative stress, apoptosis, MMP13 expression and activity, as well as a decline in autophagy. All these age-related changes partially explain the significant predisposition of aged joints to degeneration and development of OA. In addition to basal mitochondrial ROS synthesis, ROS are also synthesized by the activated receptor for advanced glycation end-products (RAGE) through activation of NAD(P)H oxidase. ROS directly damage proteins, activate p38 MAPK-induced apoptosis and NF-κB-induced cartilage breakdown *via* upregulation of ADAMTS-5 and MMP13, suggesting the importance of oxidative damage and the IL-1 pathway in initiating the age-related changes that lead to the development of OA. In support of this mechanism, a recent study has revealed the involvement of double-stranded RNA-dependent protein kinase R (PKR) in regulating p38 MAPK and p53-dependent destruction of Akt, resulted in aberrant mitochondrial biogenesis and increased oxidative stress in chondrocytes (Ma et al., 2019). Predictions by computational modeling, show the inhibitory effect of blocking the IL-1 pathway on MMP13 production and inhibition of ALK1-mediated MMP-13 synthesis in the amelioration of cartilage degeneration of aged cartilage. These latter studies establish a firm evidential basis for therapeutic interventions (Hui et al., 2016).

### Telomere Shortening and Telomerase Dysfunction

In human articular chondrocytes, the average rate of telomere shortening is approximately 40 base pairs/year (Martin and Buckwalter, 2001) Telomeres maintain chromosome stability by preventing chromosomal end fusion, and in embryonic stem cells telomeres are enzymatically renewed through the activity of telomerase (Kuszel et al., 2015). Most somatic cells lack detectable telomerase activity and so are susceptible to telomere shortening (Greider, 1998). Cellular stress can reactivate inactive telomerase gene expression, leading to telomere extension and the reacquisition of genomic stability; unregulated reactivation of telomerase can also lead to malignant transformation (Mollano et al., 2002). An analysis of telomeres in equine articular chondrocytes has shown that telomerase activity decreases with advancing age and telomerase activity is present in prepubescent horses but not postpubescent horses, implying that telomerase-positive chondrocytes from prepubescent donors are superior for cartilage repair approaches. Furthermore, it was found that while anabolic stimuli do not affect prepubescent telomerase activity, catabolic stimuli diminishes it (Wilson et al., 2014). In general, chondrocyte chromosomal telomere shortening is positively associated with biological aging and pathogenesis of OA (Martin and Buckwalter, 2001; Tamayo et al., 2011). In addition to the mean telomere length of cells, critically short telomeres appear to have a disproportionate influence on cell viability and fate (Kuszel et al., 2015). In 2012, Harbo et al. (2013) documented that the mean telomere length and the appearance of ultra-short telomere (below 1,500 basepairs) correlate with OA severity, proximity to lesions, and senescence level. The direct relationship between ultra-short telomeres and biological aging has, however, yet to be fully elucidated. A gradual reduction of mean telomere length reflects replicative senescence whereas the presence of ultra-short telomere is suggestive of stress-induced senescence. Therefore, ultra-short telomeres are potential biomarkers of oxidative damage and their presence is indicative of cellular senescence (Maria Harbo et al., 2012). OA is believed to be an accelerated local aging disease associated with premature articular cartilage senescence, and, shortened telomeres and increased chromosomal aberrations in chondrocytes can contribute to locally advanced senescence (Fragkiadaki et al., 2020). Generalized increases in genomic instability lead to an accelerated systemic senescent phenotype, as shown by the increased numerical chromosomal aberrations in peripheral blood leukocytes from OA individuals that possibly enhance the age-related degenerative joint disease (Tamayo et al., 2011). Telomeres shortening by oxidative stress may be clinically important in the early diagnosis and prognosis of OA, and understanding its relationship with other metabolic factors holds a great promise in developing therapeutic targeting of chondrocytes and related disorders.

### Chondrocyte Senescence

Human articular chondrocytes can become senescent with advancing age especially following trauma and decreased cellular homeostasis of critical cellular pathways such as autophagy (Martin and Buckwalter, 2001). Two different mechanisms of senescence are suggested in chondrocytes: replicative senescence and stress-induced premature senescence (Rim et al., 2020). Upregulation of expression of inflammatory cytokines and cell cycle arrest-related genes such as interleukin-1 beta (*IL-1*β), *p16*, *p21*, *p53*, and *p38* MAPK induces senescence directly (Vinatier et al., 2018), while downregulation of chondrocyte phenotypic maintenance genes such as *SOX9*, *BMP-2*, *IGF-1*, and *TGF-*β induces senescence indirectly. *In vivo* research is required to support these concepts and only then can articular cartilage regeneration strategies be developed to overcome the current impediments to tissue repair (Ashraf et al., 2016). Various types of cell-intrinsic and cell-extrinsic stress stimuli activate cellular senescence program orchestrated by the interplay of various cellular signaling cascades which eventually activate cell cycle arrest/senescence regulators, either p53 or p16 or both (van Deursen, 2014). Cell senescence *via* activation of p53-p21-pRb pathway can be reversed by inactivation of p53 or oncogenic Ras; p53-inactivated cells resume extensive proliferation culminating in crisis, whereas oncogenic Ras resumes limited cell proliferation. Once cells fully engage the p16-pRb pathway, subsequent inactivation of p53 and pRb, as well as silencing of p16, stimulates DNA synthesis (S phase) which lead to failure in activation of proliferation, indicating permanent cell cycle arrest. This evidence suggests that p16 is essential for establishing the irreversibility of senescence (Beauséjour et al., 2003). Senescence induction is regulated by many signaling pathways including p38MAPK/NF-κB pathways and Akt signaling that hamper the integrity of articular cartilage. In general, Akt can transduce both proanabolic and procatabolic signaling in response to diverse stimuli during cartilage repair (Greene and Loeser, 2015) exemplified by *PTEN*-deficient articular chondrocytes that exhibit high levels of senescence inducers p16^Ink4a^ and p53, senescence-associated β-galactosidase activity, and typical features of a SASP (Xie et al., 2019).

As discussed earlier, telomeres shorten with the chronological age of chondrocyte donors and telomere changes are associated with senescence-like phenotypic drift (Martin and Buckwalter, 2001; Musumeci et al., 2015). Owing to the postmitotic nature of articular cartilage where chondrocyte renewal is virtually absent, stress-induced shortening of telomere is more likely than replicative shortening of telomeres. Stimuli including excessive mechanical loading, inflammation, and persistent oxidative stress cause an increased level of ROS which leads to DNA, protein, lipid and organelle damage. DNA damage induces telomere shortening that impacts the Hayflick limit, i.e., the ability of cells to re-enter the cell cycle for further rounds of cell division ultimately leads to cellular senescence and that propagation of senescence leads to cell death (Musumeci et al., 2015). Senescent chondrocytes arrest in the G1 phase of the cell cycle secret SASP, in which accumulation of the SASP-expressing cells contributes to tissue senescence by impairing the ECM attributed to the increased production of degradative enzymes, MMPs. Moreover, aging and/or OA-related decline in the anabolic and proliferative response to growth factors as well as the loss of cellularity support the concept that chondrocyte senescence contributes to the progression of cartilage degeneration (Musumeci et al., 2015). Apart from biological aging, *in vitro* serial expansion (four passages) of chondrocytes in monolayer culture reported to turn on the senescence- and dedifferentiation-mediated genes, leading to the loss of cartilage regeneration ability (Ashraf et al., 2016). Taking all into account, the association between aging/trauma, senescence, and phenotypic changes reduce the number of healthy and functioning chondrocytes, hence promoting cartilage degeneration and eventually lead to osteoarthritic pathophysiology.

### Reduced Growth Factor Response

In articular cartilage, several growth factors are known to modulate signaling pathways involved in the stimulation of cellular quiescence, growth, division, and differentiation, hence regulating the development and homeostasis of cartilage. It is executed *via* multiple modes including the level of receptors, the concentration of growth factor ligands and growth factors. TGF-β signals *via* the ALK5 receptor and maintains young chondrocytes in a quiescent state however, the level of ALK5 receptor declines with age leading to an increased ratio of ALK1–ALK5. Despite the protective role of TGF-β under normal physiological condition, enhanced signaling *via* ALK1 in aged chondrocytes leads to an upregulation of MMP-13, thereby initiating homeostatic imbalance and cartilage breakdown (Hui et al., 2016). Insulin growth factor 1 (IGF-1) has been shown to have anabolic effects in cartilage under normal circumstances, and decreased levels of IGF-1 also play a critical role in switching the balance toward catabolic metabolism during the development of OA (Wei et al., 2017). Under conditions of oxidative stress, IGF-1 does not promote chondrocyte survival (Del Carlo and Loeser, 2003). Given that chondrocyte responsiveness to growth factor stimulation decreases with age (Loeser et al., 2002), the effect of increased oxidative stress in decreasing the survival-promoting capacity of IGF-1 is amplified (Del Carlo and Loeser, 2003). Excessive levels of ROS have been found to inhibit activation of the IRS-1/PI3K/Akt signaling pathway, which normally promotes matrix synthesis, while at the same time ROS activates the ERK MAP kinase which suppresses aggrecan, type II collagen, and Sox-9 expression. Sustained activation of ERK is associated with cell senescence, and a study using rat chondrosarcoma cells has shown that sustained ERK activation, mediated by FGFR3, promoted the expression of markers that are involved in the senescent phenotype. Extracellular ROS also contribute to the inhibition of the Akt pathway through oxidized low-density lipoproteins (LDL). Oxidized LDL binding to LOX-1 has been found to induce chondrocyte senescence which was associated with reduced levels of Akt phosphorylation after IGF-1 stimulation. Oxidative stress induced by oxidized LDL has also been associated with the promotion of the hypertrophic chondrocyte phenotype which has been described in OA cartilage (Loeser, 2011). It is intriguing to understand how aging intertwines with the expression of growth factor receptors which is implicated in survival and the response level of the cells to stimulatory and inhibitory signals to modulate their activities. Current efforts in this line is crucial in paving a path for new improved interventions not only to treat aging associated cartilage conditions but also to provide prevention strategies for healthy aging, and this is discussed in the later section on therapeutic opportunities.

### Epigenetics

It is proposed that genetic factors determine the 20–30% of the variation in human lifespan whereas non-genetic factors, stochastic events, and environment determine the remaining 70–80% of the variation. Stochastic events and environmental factors lead to epigenetic modifications, and these are a major contributor to the aging phenotype. During aging, mammalian cells undergo extensive epigenetic changes, resulting in global DNA hypomethylation and promoter hypermethylation (Muñoz-Najar and Sedivy, 2011). It has been suggested that the global DNA hypomethylation during aging is likely the outcome of the passive demethylation of heterochromatic DNA caused by a progressive loss of DNA (cytosine-5)-methyltransferase 1 (DNMT1) function and/or erroneous targeting of this enzyme by other cofactors. Genomic DNA hypomethylation possibly leads to an overexpression of *de novo* factors. DNA methylase DNMT3b could lead to DNA methylation, resulting in aberrant hypermethylation of promoter CpG islands of many genes that are initially unmethylated. The role of epigenetics in linking aging and OA is still an emerging and promising field. Promoter hypermethylation of estrogen receptor and insulin-like growth factor II (IGF2) during aging predispose elderly to sporadic colorectal tumorigenesis (Muñoz-Najar and Sedivy, 2011), similarly, promoter hypermethylation of estrogen receptor and IGF2 may cause a deficiency of estrogen and IGF2 in maintaining articular cartilage, thereby accelerating cartilage turnover and predispose the elderly (and in particular females) to develop OA. However, there is no experimental evidence supporting a direct relationship between hypermethylation of these genes during aging and OA. On the other hand, epigenetic mechanisms could mediate aberrant gene expression of transcription factors, cytokines, ECM degradative enzymes, and ECM proteins in articular chondrocytes, thus triggering the onset of OA. It has been documented that DNA methylation and histone acetylation can mediate the downregulated expression of SOX9 in advanced OA. The question whether epigenetically modified expression of SOX9 in articular cartilage is the cause or the result of OA has yet to be answered (M. Zhang and Wang, 2015). Upregulation of microRNAs miRNA-199a-3p and miRNA-193b with age may be involved in the chondrocyte senescence by downregulating anabolic factors such as SOX9, aggrecan, and collagen type II. Conversely, downregulation of miRNA-320c expression with age may be involved in the juvenile-like phenotypic properties of chondrocytes by downregulating catabolic factor ADAMTS-4. These findings suggest that miRNA-199a-3p, miRNA-193b, and miRNA-320c could be functional markers of cartilage degeneration and evaluation of donor tissues for cartilage grafting (Ukai et al., 2012).

### Metabolism

Macroanatomically, chronic metabolic and oxidative stress lead to a thinning of the collagen and proteoglycan layers as well as disorganization of collagen fiber orientation. Microanatomically, superficial, transitional, and radial zones of cartilage exhibit a loss of chondrocytes and ECM proteins (Hardin et al., 2015). Subchondral ischemia resulting from hypertension associates with OA compromising nutrient exchange into articular cartilage, hence prompting bone remodeling. Ectopic lipid deposition in cartilage induced by dyslipidemia might initiate the development of OA, impaired by deregulated cellular lipid metabolism in joint tissues. Hyperglycemia accelerates oxidative stress and AGE product formation which are implicated in cartilage degeneration, whereas low-grade systemic inflammation contributes to a degenerative internal cartilage niche that leads to OA progression OA. Obesity-related metabolic factors, particularly altered levels of adipokines lead to the expression of various proinflammatory factors and degradative enzymes, leading to the inhibition of cartilage matrix production, simultaneously spur remodeling of subchondral bone (Zhuo et al., 2012).

### Modulation in ECM Components

The half-life of type II collagen on average is approximately 100 years (Verzijl et al., 2000) and aggrecan 3.5 years (Maroudas et al., 1998). In contrast to the whole aggrecan protein, the aggrecan G1 domain with the role of hyaluronic acid binding has a lower turnover rate of approximately 25 years. Aggrecan turnover gives rise to proportionately more G1 domain fragments occupying hyaluronic acid, therefore, inferior aggrecan structures are generated upon aging (Hardin et al., 2015). Age-related changes not only occur in chondrocytes but also in the cartilage matrix, thereby contributing to OA development. MRI studies show that knee cartilage thinning occurs during aging, particularly on the femoral side of the joint and on the patellae, suggesting a gradual loss of cartilage matrix with age. Thinning can be due to the loss of chondrocytes and/or reduced growth factor responsiveness but also in part to something as simple as reduced water content. Excessive collagen cross-linking, visualized by yellowing of tissue through glycation, increases cartilage stiffness and brittleness, thereby increasing susceptibility to fatigue failure. Increased levels of AGEs in cartilage is correlated with declining anabolic activity (Loeser, 2011).

In surveying the numerous molecular pathways underlying the degenerative process in OA, their relationship to senescence/aging or longevity of chondrocytes, only serves to highlight their intricacy and interdependency. Hence, understanding the complexity of these pathways and the discovery of tools targeting them that are relevant to depressing inflammation, oxidative stress, and senescence in aging chondrocytes may be important in combating, treating, or reversing chronic diseases like OA.

## Therapeutic Opportunities and Future Perspective

Degeneration of cartilage is considered to be a multifactorial dysregulation of cellular systems, where the cellular processes are interlinked and regulate each other. Fundamentally, aging and degeneration of the cartilage that leads to OA is attributed by increased inflammation and decreased regenerative potential of cartilage. Ideally, therapeutics that could restore impaired function of chondrocytes and reverse/delay cartilage aging will involve the modulation of the latter two elements. We, in this review, propose the treatments to achieve this could be pharmacological interventions or/and cell-based therapy that offer suppression of excessive inflammation and support regenerative capabilities.

**FIGURE 1:**
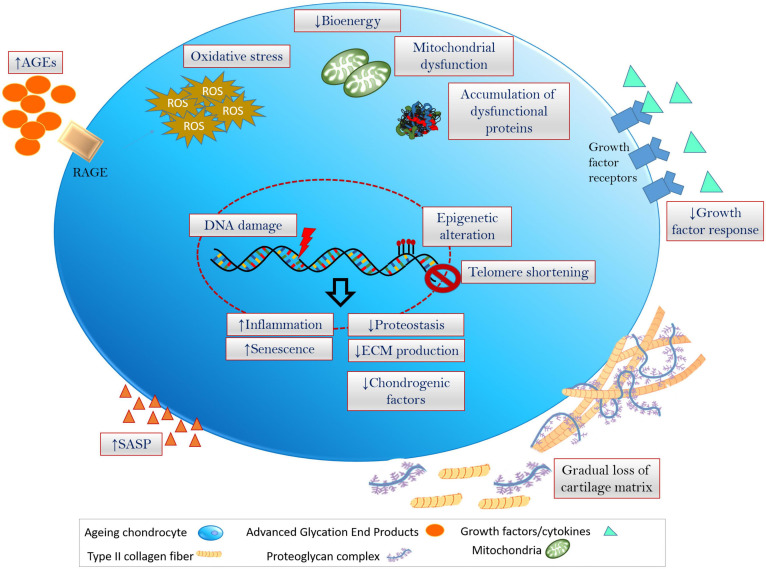
Hallmarks of chondrocyte aging inflammation, oxidative stress (excess production and accumulation of ROS and the related dysfunctioning of cellular components) and senescence are fundamental events that is associated with the onset of cellular aging and aging-associated degenerative disorders. Chondrocytes undergoing aging at multiple levels including genetic/epigenetic aberration, disruption in biochemical and bioenergetics related cellular processes, modulation of ECM and cartilage niche, leading to the degeneration of cartilage and onset of OA.

### Pharmacological Treatments

Collectively, understanding molecular determinants of chondrocyte aging and OA has paved a way in identifying potential pharmacological treatments that can regulate these deregulated pathways, hence reversing or delaying the degeneration of cartilage. Treatments including apoptosis-inducing reagents in a model of genetic apoptosis inhibition, arsenic trioxide, hypoxia, mETC inhibitors, and a short mitochondrial form of p19^ARF^ have been reported to induce autophagic cell death (Yongqiang Chen et al., 2010). Despite the promising outcomes by mTOR inhibitors in the treatment of cancer and other diseases, rapamycin (mTOR inhibitor) and its rapalogs have not been tested in OA in the clinical setting. mTOR has a negative feedback inhibition on the activity of PI3K/Akt pathway, and the inhibition of mTOR leads to increased activity of the PI3K/Akt/NF-kB pathway, which may increase MMP secretion by chondrocytes. Therefore, by simultaneously targeting the PI3K/Akt/NF-kB pathway, dual inhibition of PI3K and mTOR can be considered a potential therapeutic approach for OA (Pal et al., 2015). Pharmacological upregulation of autophagy by rapamycin reduces the severity of experimental OA, synovitis, ADAMTS-5, and IL-1β expression, thus enhancing chondrocyte survival and preventing glycosaminoglycan loss. Though these results are encouraging, the potent antiproliferative and immunosuppressive effects of rapamycin pose an additional challenge in translating such strategies for human applications. Nutrient supplementation with non-immunosuppressive compounds such as spermidine, polyamines, or ω-6 polyunsaturated fatty acid or treatment with activators of the UPS could be considered, yet the safety, specificity, and efficacy need to be experimentally and clinically validated. Glucosamine is a safe and widely used dietary supplement that has the potential in enhancing cartilage health in human, and also acts as an effective activator of autophagy. Glucosamine activates autophagy *in vitro* and *in vivo via* the Akt/Fox03/mTOR signaling pathways, raising the feasibility that glucosamine can be utilized to maintain cellular homeostasis and joint health although such treatment needs to be continual and long term to be beneficial (Goldring and Berenbaum, 2015). Disease and pain control with glucocorticoid therapy in OA has been employed extensively for decades, acting by diffusing across cellular membranes, binding their cognate nuclear receptors and, interrupting the inflammatory and immune pathways at a number of levels. A recent study demonstrated that dexamethasone, a synthetic glucocorticoid, increases the intracellular ROS levels, autophagy markers, and expression of FoxO3. In response to the increased ROS level, autophagy is induced as a defense mechanism *via* ROS/Akt/FoxO3 pathway which subsequently protects human chondrocytes from ROS-induced apoptosis. Of note, long-term administration of dexamethasone increasing ROS level could upregulate the expression of ADAMTSs and MMPs *via* ROS-dependent manner, thereby contributing to advancing cartilage degeneration (Shen et al., 2015). There have been many studies that have pursued the targetting of these metabolic pathways in order to stabilise or reverse OA disease especially using natural products, nutrients, pharmacological agents and biologics such as cell-free and cell-based regenerative strategies, and a selection of these are captured in Table 1.

**TABLE 1:** Chondroprotective therapeutics and the underlying mechanism of action targeting aging and degeneration associated determinants.

**Categories of therapeutic strategies**	**Therapeutics**	**Therapeutic mechanism**	**References**
Natural products/nutrients and their derivatives	Ascorbic acid	• Protection for human chondrocytes against oxidative stress	Chang et al., 2015
	Baicalin	• Prevented the apoptosis of endplate chondrocytes by inhibiting the oxidative stress • Inhibited endoplasmic reticulum stress •Protects human OA chondrocytes against IL-1β-induced apoptosis •Protects the degradation of ECM through activating autophagy via miR-766-3p/AIFM1 axis	Pan et al., 2017; Cao et al., 2018; Li et al., 2020
	Curcumin	• Protected the mitochondrial function, hence prevented cartilage degeneration •improves age-related and surgically induced osteoarthritis by promoting autophagy •Inhibited apoptosis of chondrocytes through activation ERK1/2 signaling Pathways induced autophagy •Inhibited the PERK-eIF2α-CHOP pathway through promoting SIRT1 expression in oxidative stress	Li et al., 2017; Feng et al., 2019; Nicoliche et al., 2020
	Delphinidin (a primary plant pigment, and also an antioxidant)	• Cytoprotects chondrocytes against oxidative stress through activation of autophagy	Lee et al., 2020
	Diosmin	• Chondroprotective effect via modulating oxidative stress	Yi−Ru Chen et al., 2019
	Polyphenols derived by olive extracts (e.g., Oleuropein)	Targeted Cx43 and senescence	Varela-Eirín et al., 2020
	Resveratrol	• Exerted anabolic, anti-catabolic, anti-inflammatory and chondroprotective effects •Delays cartilage degeneration autophagy via AMPK/mTOR pathway	Im et al., 2012; Qin et al., 2017
	Vitamin D	• Activated autophagy via mediating the AMPK–mTOR signaling pathway in chondrocytes, to reduce osteoarthritis	Kong et al., 2020
Pharmacological agents (biological factors/Drugs)	Irisin, a cleaved form of fibronectin type III domain containing 5 (FNDC5)	• Modulated Oxidative Stress •Regulated mitochondrial Integrity •Regulated autophagy	Wang et al., 2020
	Fenofibrate	• Senotherapeutic molecules with pro-autophagic activity	Nogueira-Recalde et al., 2019
	Navitoclax (ABT263)	• A specific inhibitor of the BCL-2 and BCL-xL proteins •Reduced inflammation •Senolytic drug	Yang et al., 2020
	Peroxiredoxin II (Prx II)	• Anti-oxidative stress and anti-aging effects •Reduced oxidative stress and cell senescence in chondrocytes by activating the p16-CDK4/6-pRb-E2F signaling pathway	Shao et al., 2020
	Rapamycin	• A specific inhibitor of the mTOR signaling pathway •Enhanced expression of autophagy regulators and prevents chondrocyte death.	Caramés et al., 2012; Pal et al., 2015; Bao et al., 2020
Biologics (cell-based)	Articular cartilage progenitors	• Resistance to telomere erosion through the expression of telomerase •Tissue replacement therapies	Dowthwaite, 2004; Williams et al., 2010; McCarthy et al., 2012; Jiang et al., 2016
	Adult stem cells (tissue-specific and mesenchymal stem cells)	• Reduced catabolic effect •Reduced inflammation—via their indirect regenerative effects (secretomes and EVs) •Immunomodulatory effect •Anti-apoptosis and anti-fibrosis •Tissue replacement therapies	Samuel et al., 2018; Samuel et al., 2019
	Embryonic stem cells/induced pluripotent stem cells	• Tissue replacement therapies	Chang et al., 2020; Gardner et al., 2019
Biologics (cell-free)	Platelet-rich plasma	• Reduced inflammation •Regulates cell chemotaxis •Improved angiogenesis •Enhanced cell proliferation and cell differentiation •Enhanced ECM production, hence matrix deposition	Moussa et al., 2017; Garbin and Olver, 2020
	Extracellular vesicles/exosomes	• Improved cartilage thickness •Increased matrix deposition •Better subchondral bone integrity •Reduced synovial cell apoptosis •Reduced MMPs	Wang et al., 2017; Khatab et al., 2018; Zhang et al., 2019; Jin et al., 2020

The use of ROS scavengers is probably the simplest strategy to prevent stress-induced senescence; antioxidants, ascorbic acid (vitamin C), *N*-acetylcysteine, sodium pyruvate, sodium selenite, and Trolox (*see also* Table 1) improve mesenchymal stem cell cellular “health,” increase cell yield, and maintain the differentiation potential of cells (Turinetto et al., 2016). RNA interference of p16^Ink4a^ in OA cell cultures can restore their anabolic metabolic responsiveness to growth factors, similar to younger fetal chondrocytes, but the effect appears to require continual treatment to suppress p16^Ink4a^ expression (Zhou, 2004). To prove the causal role of senescent cells in chronic disease, Baker et al. (2011) developed an *in vivo* transgene model to selectively remove p16^Ink4a^ +ve senescent cells by apoptosis in aged and prematurely aging hypomorphic BubR1^*H*/H^ mice which then showed delayed onset of sarcopenia, prevention of adipose loss and cataract formation. Baar et al. (2017) used cell-penetrating peptides that target FOXO4 interactions with p53 localized at DNA segments with chromatin alterations reinforcing senescence (DNA-SCARS) to sensitize senescent cells to p53-dependent apoptosis. FOXO4-p53-interfering peptides can be used to induce apoptosis in senescent cells *in vitro* and *in vivo*, in the latter context reducing fragility and renal failure in fast aging Xpd^*TTD/TTD*^ and naturally aging mice. Similarly, dasatinib and quercertin also function in combination to eliminate senescent cells and reduce frailty in aged mice through inhibition of Src kinase and antiapoptotic Bcl-xL (Xu et al., 2018). The use of dasatinib/quercertin to treat OA has been mooted (McCulloch et al., 2017), but their use *in vitro* to prepare cells for ACI is possibly a more targeted application. Despite convincing data pointing to the causal role of senescence in chronic conditions, in light of the evidence demonstrating senescent cell participation in promoting wound healing (Demaria et al., 2014), stem cell priming and plasticity (Ritschka et al., 2017), and limb regeneration (Yun et al., 2015), aspects of their physiological function has to be appraised. In cartilage, the accumulation of senescent cells in adult tissues may help to retain cellularity in a tissue that otherwise could be depopulated below a threshold level for maintenance of the ECM.

Evaluation of recent studies suggests the secretory profile of senescent cells constitutes a transient signal to initiate repair processes, while persistent activation of a proinflammatory secretome is the basis of chronic wounding and disease (Kowald et al., 2020). Again, these are reasons why senolytic interventions may be best suited to generating a stable, progenitor population for cell therapy rather than treating OA joints.

### Regenerative Therapies

#### Cell-Based Therapies

Pharmacological treatments to regulate pathways leading to accelerated aging of chondrocytes in cartilage can potentially reduce the severity of OA in patients with established disease affecting the whole joint. This approach is less amenable for younger patients who at first presentation are symptomatic with localized cartilage lesions in their joints. These lesions can be repaired using a variety of cell therapies that overcome an inherent barrier to cellular migration facing chondrocytes embedded within a dense ECM at the wound edges (Hunziker, 2002). For example, mesenchymal stem cells (MSC) in the marrow beneath the overlying subchondral bony plate can be released into the lesion by drilling or puncturing through the plate. However, microfracture produces transient fibrocartilaginous repair tissue that is functionally suboptimal.

Another, no less-invasive procedure, autologous chondrocyte implantation (ACI), requires two surgeries, the first to remove a cartilage biopsy, from which cells are cultivated, that are then transplanted in a second procedure into the debrided lesion under a periosteal flap (Brittberg et al., 1994). The latter procedure produces more hyaline cartilage than microfracture. The inherent disadvantage of ACI is the number of cells isolated and expanded from the biopsy limits the size of the lesion that can be repaired because the ability of cells to efficiently redifferentiate reduces markedly upon >5 passages in culture (Schulze-Tanzil et al., 2002). Also, replicative senescence within the expanded cell population may also further limit their repair potential. To overcome these limits, repair strategies have progressed to use allogeneic mesodermal (and tissue-specific) progenitors and adult stem cells/MSCs; as allogeneic transplanted cells differentiate and become embedded in a supportive ECM, they are effectively immune privileged.

As mentioned earlier, mature chondrocytes have limited ability to repair cartilage defects due to an inherent inability to migrate through the ECM, one approach to repair cartilage defects is by introducing a new cell population to stimulate repair and produce structural repair of lesions. Hence, MSCs are considered to be an excellent compatible cellular source that are easily expanded in culture, and following seeding in an artificial matrix, can be implanted into a cartilage defect and retain the capacity to undergo chondrogenesis and generate hyaline cartilage. Additionally MSCs can also be used to produce paracrine factors to induce cartilage repair, either alone or implanted in combination with autologous articular chondrocytes (Sariset al., 2021).

*In vitro* expanded tissue-specific articular cartilage progenitors exhibit resistance to telomere erosion through the expression of telomerase, and, in contrast to bone marrow-derived MSCs, they preferentially differentiate to form hyaline cartilage rather than calcified cartilage or bone (Dowthwaite, 2004; Williams et al., 2010; McCarthy et al., 2012). Cell kinetic and telomeric analysis of articular chondroprogenitors from normal and OA human cartilage show approximately 50% of OA progenitors undergo accelerated senescence following culture expansion (Fellows et al., 2017). Zhou (2004) discovered OA chondrocytes show a higher trend of p16^Ink4a^ expression than age-matched controls and fetal cartilage, and, that this pattern of expression extends to cells following isolation and culture expansion. These data argue for the isolation of chondroprogenitors from younger non-diseased donors for culture expansion (Adkisson et al., 2010). Senolytic or senostatic molecules can be used to maintain a healthy viable progenitor population during an *in vitro* cell culture expansion phase, removing cells that would otherwise compromise repair through “bystander” effects upon transplantation.

### Cell-Free-Based Regenerative Therapies

#### Platelet-Rich Plasma-Based Therapy

In recent years, much effort has also been directed to study the therapeutic value of naturally occurring biomolecule pools such as platelet-rich plasma (PRP) for cartilage regeneration. The presence of many important growth factors in PRP may enhance the anabolic signal for regeneration, and thus may offer therapeutic benefit to patients with OA (Marmotti et al., 2015). Applying a similar theoretical framework, PRP may also enhance cellular expansion and chondrogenesis of the MSCs, thus may synergistically improve cartilage repair. The reproducible positive effect of PRP on chondrocyte and MSC proliferation and chondrogenic differentiation indicates that the adjunct of use of PRP may be advantageous to promote cellular expansion *in vitro* for the enhancement of cell-based therapy.

The regenerative capacity of PRP is mainly attributed to its broad biomolecular composition, including chemokines, cytokines, small molecules, adhesive proteins, proteases, antiproteases, exosome-derived microRNAs, receptor ligands, and growth factors, all of which are essential components for the initiation and maintenance of the tissue healing response (Garbin and Olver, 2020). This includes regulation of cell chemotaxis, angiogenesis, cell proliferation, cell differentiation, and ECM production (Foster et al., 2009; Gobbi et al., 2012; De La Mata, 2013). Multiple clinical studies have shown that intra-articular injection of PRP significantly ameliorates OA symptoms (Kon et al., 2010; Filardo et al., 2011; Gobbi et al., 2012; Patel et al., 2013; Raeissadat et al., 2013). Intriguingly, the use of PRP has been shown in immature bovine cartilage explants to induce articular cartilage maturation including reorganization of the ECM into a more adult-like state and this may underly some of the efficacy noted for PRP injections especially in younger patients (Zhang et al., 2017).

#### Extracellular Vesicles as an Emerging Therapeutic Approach

The relevance of extracellular vesicles (EVs) in regulating the development of age-related conditions is based on the notion that EVs are one of the known mechanisms responsible for the maintenance of cellular homeostasis (Baixauli et al., 2014; Desdín-Micó and Mittelbrunn, 2017). Loss of EV regulatory influence contributes to the deregulation of processes essential for cellular integrity and signaling pathways involved in cellular metabolism and growth (Yafei Wang et al., 2017). Consequently, this leads to the development of cellular events such as oxidative stress, protein aggregation, mitochondrial dysfunction, and inflammation (Klaips et al., 2018), all of which are contributing factors of aging, as discussed earlier. Intriguingly, stem cells, as somatic cells, release a large number of EVs (Drago et al., 2013; Katsuda et al., 2013; Tetta et al., 2013). The role of EVs in mediating tissue repair by stem cells from which they are derived has been consistently demonstrated (Camussi et al., 2010; Ratajczak, 2011; Panagiotou et al., 2016).

Preclinical studies especially *in vivo* studies revealed injection of secretomes or EVs derived from multiple cellular sources improves cartilage thickness, matrix deposition, and subchondral bone integrity, reduced synovial cell apoptosis, and reduced MMPs in animal injury models (Yafei Wang et al., 2017; Khatab et al., 2018; Zhang et al., 2019; Jin et al., 2020).

While these *in vivo* studies directly injected EVs intra-articularly, their study designs were highly varied. Moving forward, clinical studies for EV transplantation requires deliberate consideration on the standardization of EV preparation protocol, dose, and injection times, thus allowing more reproducible and comparable datasets to be used to progress treatments for cartilage degeneration in OA.

## Conclusion

In summary, cellular homeostasis of chondrocytes and cartilage is maintained through the molecular sensors regulating complex yet interlinked cellular events including bioenergetical homeostasis, survival, the balance of oxidative and antioxidative production, genetic integrity, mechanobiology, and intercellular communications within the tissue niche. When deregulation occurs in tissue and cellular homeostasis of cartilage, it leads to degenerative disorders. In this review, we have highlighted the hallmarks of chondrocyte aging and degeneration of cartilage in light of their key molecular determinants and their underlying mechanisms. Recent efforts in developing therapeutics that target deregulated cellular homeostasis are captured too. Moving forward, novel approaches for activating the deregulated survival pathways and restore the balance of homeostasis through naturally occurring nutrients and natural products or pharmacological interventions or even innovative strategies using biologics for slowing down or reversing aging of cartilage should be further investigated. This new generation of treatment strategies can potentially make a significant impact on improving the lives of patients suffering from many aging associated chronic diseases like OA.

## Author Contributions

TSR conceptualized the scope of the manuscript. TSR, YMY, and IMK drafted the manuscript. All authors approved the final manuscript.

## Conflict of Interest

The authors declare that the research was conducted in the absence of any commercial or financial relationships that could be construed as a potential conflict of interest.

## Abbreviations

ATP: adenosine triphosphate
AGEs: advanced glycation end products
AMPK: AMP-activated protein kinase
ACI: autologous chondrocyte implantation
DNMT1: DNA (cytosine-5)-methyltransferase 1
ECM: extracellular matrix
EVs: extracellular vesicles
ER: endoplasmic reticulum
FOXO3: forkhead family O subclass transcription factor 3
*IL-1*β: interleukin-1 beta
LDL: low-density lipoproteins
mtDNA: mitochondrial DNA
MRC: mitochondrial respiratory chain
TFAM: mitochondrial transcription factor A
MAPK: mitogen-activated protein kinase
mTOR: mammalian target of rapamycin
NF-κB: nuclear factor-κ B
NO: nitric oxide
OA: osteoarthritis
PGC-1α: peroxisome proliferator-activated receptor g coactivator 1α
PTEN: phosphatase and tensin homolog deleted on chromosome 10
ROS: reactive oxygen species
RAGE: receptor for advanced glycation end-products
SASP: senescence associated secretory phenotype
Sirt1: Sirtuin 1
UPS: ubiquitin-proteosome system.

## References

[B1] Adkisson H. D. IV Martin J. A. Amendola R. L. Milliman C. Mauch K. A. Katwal A. B. (2010). The potential of human allogeneic juvenile chondrocytes for restoration of articular cartilage. *Am. J. Sports Med.* 38 1324–1333. 10.1177/0363546510361950 20423988 PMC3774103

[B2] Ansari M. Y. Ahmad N. Voleti S. Wase S. Malik M. Novak K. (2020). Mitochondrial dysfunction in osteoarthritis and aged cartilage triggers inflammatory response and matrix degradation via ros mediated activation of JNK-MAPK/cFos-AP1 axis in chondrocytes. *Osteoarthritis Cartilage* 28:S187. 10.1016/j.joca.2020.02.304

[B3] Ashraf S. Cha B.-H. Kim J.-S. Ahn J. Han I. Park H. (2016). Regulation of senescence associated signaling mechanisms in chondrocytes for cartilage tissue regeneration. *Osteoarthritis Cartilage* 24 196–205. 10.1016/j.joca.2015.07.008 26190795

[B4] Baar M. P. Brandt R. M. C. Putavet D. A. Klein J. D. D. Derks K. W. J. Bourgeois B. R. M. (2017). Targeted apoptosis of senescent cells restores tissue homeostasis in response to chemotoxicity and aging. *Cell* 169 132–147.e16. 10.1016/j.cell.2017.02.031 28340339 PMC5556182

[B5] Baixauli F. López-Otín C. Mittelbrunn M. (2014). Exosomes and autophagy: coordinated mechanisms for the maintenance of cellular fitness. *Front. Immunol.* 5:403. 10.3389/fimmu.2014.00403 25191326 PMC4138502

[B6] Baker D. J. Wijshake T. Tchkonia T. LeBrasseur N. K. Childs B. G. van de Sluis B. (2011). Clearance of p16Ink4a-positive senescent cells delays ageing-associated disorders. *Nature* 479 232–236. 10.1038/nature10600 22048312 PMC3468323

[B7] Bao J. Chen Z. Xu L. Wu L. Xiong Y. (2020). Rapamycin protects chondrocytes against IL-18-induced apoptosis and ameliorates rat osteoarthritis. *Aging* 12 5152–5167. 10.18632/aging.102937 32182210 PMC7138594

[B8] Beauséjour C. M. Krtolica A. Galimi F. Narita M. Lowe S. W. Yaswen P. (2003). Reversal of human cellular senescence: roles of the p53 and p16 pathways. *EMBO J.* 22 4212–4222. 10.1093/emboj/cdg417 12912919 PMC175806

[B9] Blanco F. J. Rego I. Ruiz-Romero C. (2011). The role of mitochondria in osteoarthritis. *Nat. Rev. Rheumatol.* 7 161–169. 10.1038/nrrheum.2010.213 21200395

[B10] Brittberg M. Lindahl A. Nilsson A. Ohlsson C. Isaksson O. Peterson L. (1994). Treatment of deep cartilage defects in the knee with autologous chondrocyte transplantation. *N. Engl. J. Med.* 331 889–895. 10.1056/nejm199410063311401 8078550

[B11] Camussi G. Deregibus M. C. Tetta C. (2010). Paracrine/endocrine mechanism of stem cells on kidney repair: role of microvesicle-mediated transfer of genetic information. *Curr. Opin. Nephrol. Hypertens.* 19 7–12. 10.1097/MNH.0b013e328332fb6f 19823086

[B12] Cao J. Zhang Y. Wang T. Li B. (2018). Endoplasmic reticulum stress is involved in baicalin protection on chondrocytes from patients with osteoarthritis. *Dose Response?* 16:1559325818810636. 10.1177/1559325818810636 30505248 PMC6256307

[B13] Caramés B. Hasegawa A. Taniguchi N. Miyaki S. Blanco F. J. Lotz M. (2012). Autophagy activation by rapamycin reduces severity of experimental osteoarthritis. *Ann. Rheum. Dis.* 71 575–581. 10.1136/annrheumdis-2011-200557 22084394 PMC3294168

[B14] Caramés B. Taniguchi N. Otsuki S. Blanco F. J. Lotz M. (2010). Autophagy is a protective mechanism in normal cartilage, and its aging-related loss is linked with cell death and osteoarthritis. *Arthritis Rheum.* 62 791–801. 10.1002/art.27305 20187128 PMC2838960

[B15] Chang J. U. N. Wang W. E. I. Zhang H. U. I. Hu Y. Wang M. Yin Z. (2013). The dual role of autophagy in chondrocyte responses in the pathogenesis of articular cartilage degeneration in osteoarthritis. *Int. J. Mol. Med.* 32 1311–1318. 10.3892/ijmm.2013.1520 24126970

[B16] Chang Y. H. Wu K. C. Ding D. C. (2020). Induced pluripotent stem cell-differentiated chondrocytes repair cartilage defect in a rabbit osteoarthritis model. *Stem Cells Int.* 2020:8867349. 10.1155/2020/8867349 33224204 PMC7671807

[B17] Chang Z. Huo L. Li P. Wu Y. Zhang P. E. I. (2015). Ascorbic acid provides protection for human chondrocytes against oxidative stress. *Mol. Med. Rep.* 12 7086–7092. 10.3892/mmr.2015.4231 26300283

[B18] Chen Y. Azad M. B. Gibson S. B. (2010). Methods for detecting autophagy and determining autophagy-induced cell deathThis review is one of a selection of papers published in a Special Issue on Oxidative Stress in Health and Disease. *Can. J. Physiol. Pharmacol.* 88 285–295. 10.1139/y10-010 20393593

[B19] Chen Y. Yang K. Lu D. Wu W. Wang C. Tsai M. (2019). The chondroprotective effect of diosmin on human articular chondrocytes under oxidative stress. *Phytother. Res.* 33 2378–2386. 10.1002/ptr.6425 31270886

[B20] De La Mata J. (2013). Plasma rico en plaquetas: ?un nuevo tratamiento para el reumatólogo? *Reumatol. Clin.* 9 166–171. 10.1016/j.reuma.2012.05.011 22902984

[B21] Del Carlo M. Loeser R. F. (2003). Increased oxidative stress with aging reduces chondrocyte survival: correlation with intracellular glutathione levels. *Arthritis Rheum.* 48 3419–3430. 10.1002/art.11338 14673993

[B22] Demaria M. Ohtani N. Youssef S. A. Rodier F. Toussaint W. Mitchell J. R. (2014). An essential role for senescent cells in optimal wound healing through secretion of PDGF-AA. *Dev. Cell* 31 722–733. 10.1016/j.devcel.2014.11.012 25499914 PMC4349629

[B23] Desdín-Micó G. Mittelbrunn M. (2017). Role of exosomes in the protection of cellular homeostasis. *Cell Adh. Migr.* 11 127–134. 10.1080/19336918.2016.1251000 27875097 PMC5351736

[B24] Dowthwaite G. P. (2004). The surface of articular cartilage contains a progenitor cell population. *J. Cell Sci.* 117 889–897. 10.1242/jcs.00912 14762107

[B25] Drago D. Cossetti C. Iraci N. Gaude E. Musco G. Bachi A. (2013). The stem cell secretome and its role in brain repair. *Biochimie* 95 2271–2285. 10.1016/j.biochi.2013.06.020 23827856 PMC4061727

[B26] Fellows C. R. Williams R. Davies I. R. Gohil K. Baird D. M. Fairclough J. (2017). Characterisation of a divergent progenitor cell sub-populations in human osteoarthritic cartilage: the role of telomere erosion and replicative senescence. *Sci. Rep.* 7:41421. 10.1038/srep41421 28150695 PMC5288717

[B27] Feng K. Ge Y. Chen Z. Li X. Liu Z. Li X. (2019). Curcumin inhibits the PERK-eIF2α-CHOP pathway through promoting SIRT1 expression in oxidative stress-induced rat chondrocytes and ameliorates osteoarthritis progression in a rat model. *Oxid. Med. Cell. Longev.* 2019:8574386. 10.1155/2019/8574386 31223428 PMC6541984

[B28] Filardo G. Kon E. Buda R. Timoncini A. Di Martino A. Cenacchi A. (2011). Platelet-rich plasma intra-articular knee injections for the treatment of degenerative cartilage lesions and osteoarthritis. *Knee Surg. Sports Traumatol. Arthrosc.* 19 528–535. 10.1007/s00167-010-1238-6 20740273

[B29] Foster T. E. Puskas B. L. Mandelbaum B. R. Gerhardt M. B. Rodeo S. A. (2009). Platelet-rich plasma: from basic science to clinical applications. *Am. J. Sports Med.* 37 2259–2272. 10.1177/0363546509349921 19875361

[B30] Fragkiadaki P. Nikitovic D. Kalliantasi K. Sarandi E. Thanasoula M. Stivaktakis P. D. (2020). Telomere length and telomerase activity in osteoporosis and osteoarthritis. *Exp. Therap. Med.* 19 1626–1632. 10.3892/etm.2019.8370 32104213 PMC7027092

[B31] Garbin L. C. Olver C. S. (2020). Platelet-Rich products and their application to osteoarthritis. *J. Equine Vet. Sci.* 86:102820. 10.1016/j.jevs.2019.102820 32067662

[B32] Gardner O. F. W. Juneja S. C. Whetstone H. Nartiss Y. Sieker J. T. Veillette C. (2019). Effective repair of articular cartilage using human pluripotent stem cell-derived tissue. *Eur. Cells Mater.* 38 215–227. 10.22203/eCM.v038a15 31688947

[B33] Gavriilidis C. Miwa S. von Zglinicki T. Taylor R. W. Young D. A. (2013). Mitochondrial dysfunction in osteoarthritis is associated with down-regulation of superoxide dismutase 2. *Arthritis Rheum.* 65 378–387. 10.1002/art.37782 23138846

[B34] Gobbi A. Karnatzikos G. Mahajan V. Malchira S. (2012). Platelet-Rich plasma treatment in symptomatic patients with knee osteoarthritis: preliminary results in a group of active patients. *Sports Health* 4 162–172. 10.1177/1941738111431801 23016084 PMC3435904

[B35] Goldring M. B. Berenbaum F. (2015). Emerging targets in osteoarthritis therapy. *Curr. Opin. Pharmacol.* 22 51–63. 10.1016/j.coph.2015.03.004 25863583 PMC4470796

[B36] Greene M. A. Loeser R. F. (2015). Function of the chondrocyte PI-3 kinase-Akt signaling pathway is stimulus dependent. *Osteoarthritis Cartilage* 23 949–956. 10.1016/j.joca.2015.01.014 25659655 PMC4444401

[B37] Greider C. W. (1998). Telomerase activity, cell proliferation, and cancer. *Proc. Natl. Acad. Sci. U.S.A.* 95 90–92. 10.1073/pnas.95.1.90 9419332 PMC34198

[B38] Harbo M. Bendix L. Bay-Jensen A.-C. Graakjaer J. Søe K. Andersen T. L. (2012). The distribution pattern of critically short telomeres in human osteoarthritic knees. *Arthritis Res. Ther.* 14 R12–R12. 10.1186/ar3687 22257826 PMC3392801

[B39] Harbo M. Delaisse J. M. Kjaersgaard-Andersen P. Soerensen F. B. Koelvraa S. Bendix L. (2013). The relationship between ultra-short telomeres, aging of articular cartilage and the development of human hip osteoarthritis. *Mech. Ageing Dev.* 134 367–372. 10.1016/j.mad.2013.07.002 23872258

[B40] Hardin J. A. Cobelli N. Santambrogio L. (2015). Consequences of metabolic and oxidative modifications of cartilage tissue. *Nat. Rev. Rheumatol.* 11 521–529. 10.1038/nrrheum.2015.70 26034834 PMC4765360

[B41] He Y. Wu Z. Xu L. Xu K. Chen Z. Ran J. (2020). The role of SIRT3-mediated mitochondrial homeostasis in osteoarthritis. *Cell. Mol. Life Sci.* 77 3729–3743. 10.1007/s00018-020-03497-9 32468094 PMC11105031

[B42] Henrotin Y. E. Bruckner P. Pujol J.-P. (2003). The role of reactive oxygen species in homeostasis and degradation of cartilage. *Osteoarthritis Cartilage* 11 747–755. 10.1016/s1063-4584(03)00150-x 13129694

[B43] Horigome Y. Ida-Yonemochi H. Waguri S. Shibata S. Endo N. Komatsu M. (2020). Loss of autophagy in chondrocytes causes severe growth retardation. *Autophagy* 16 501–511. 10.1080/15548627.2019.1628541 31203752 PMC6999621

[B44] Hui W. Young D. A. Rowan A. D. Xu X. Cawston T. E. Proctor C. J. (2016). Oxidative changes and signalling pathways are pivotal in initiating age-related changes in articular cartilage. *Ann. Rheum. Dis.* 75 449–458. 10.1136/annrheumdis-2014-206295 25475114 PMC4752670

[B45] Hunziker E. B. (2002). Articular cartilage repair: basic science and clinical progress. A review of the current status and prospects. *Osteoarthritis Cartilage* 10 432–463. 10.1053/joca.2002.0801 12056848

[B46] Im H.-J. Li X. Chen D. Yan D. Kim J. Ellman M. B. (2012). Biological effects of the plant-derived polyphenol resveratrol in human articular cartilage and chondrosarcoma cells. *J. Cell. Physiol.* 227 3488–3497. 10.1002/jcp.24049 22252971 PMC3330153

[B47] Jiang Y. Cai Y. Zhang W. Yin Z. Hu C. Tong T. (2016). Human cartilage−derived progenitor cells from committed chondrocytes for efficient cartilage repair and regeneration. *Stem Cells Transl. Med.* 5 733–744. 10.5966/sctm.2015-0192 27130221 PMC4878331

[B48] Jin Z. Ren J. Qi S. (2020). Human bone mesenchymal stem cells-derived exosomes overexpressing microRNA-26a-5p alleviate osteoarthritis via down-regulation of PTGS2. *Int. Immunopharmacol.* 78:105946. 10.1016/j.intimp.2019.105946 31784400

[B49] Katsuda T. Kosaka N. Takeshita F. Ochiya T. (2013). The therapeutic potential of mesenchymal stem cell-derived extracellular vesicles. *Proteomics* 13 1637–1653. 10.1002/pmic.201200373 23335344

[B50] Khatab S. van Osch G. J. V. M. Kops N. Bastiaansen-Jenniskens Y. M. Bos P. K. Verhaar J. A. N. (2018). Mesenchymal stem cell secretome reduces pai n and prevents carti lage damage i n a muri ne osteoarthri ti s model. *Eur. Cells Mater.* 36 218–230. 10.22203/eCM.v036a16 30398288

[B51] Klaips C. L. Jayaraj G. G. Hartl F. U. (2018). Pathways of cellular proteostasis in aging and disease. *J. Cell Biol.* 217 51–63. 10.1083/jcb.201709072 29127110 PMC5748993

[B52] Kon E. Buda R. Filardo G. Di Martino A. Timoncini A. Cenacchi A. (2010). Platelet-rich plasma: intra-articular knee injections produced favorable results on degenerative cartilage lesions. *Knee Surg. Sports Traumatol. Arthrosc.* 18 472–479. 10.1007/s00167-009-0940-8 19838676

[B53] Kong C. Wang C. Shi Y. Yan L. Xu J. Qi W. (2020). Active vitamin D activates chondrocyte autophagy to reduce osteoarthritis via mediating the AMPK–mTOR signaling pathway. *Biochem. Cell Biol.* 98 434–442. 10.1139/bcb-2019-0333 31815524

[B54] Kowald A. Passos J. F. Kirkwood T. B. L. (2020). On the evolution of cellular senescence. *Aging Cell* 19:e13270. 10.1111/acel.13270 33166065 PMC7744960

[B55] Kuszel L. Trzeciak T. Richter M. Czarny-Ratajczak M. (2015). Osteoarthritis and telomere shortening. *J. Appl. Genet.* 56 169–176. 10.1007/s13353-014-0251-8 25366419 PMC4412548

[B56] Lee D.-Y. Park Y.-J. Song M.-G. Kim D. R. Zada S. Kim D.-H. (2020). Cytoprotective effects of delphinidin for human chondrocytes against oxidative stress through activation of autophagy. *Antioxidants (Basel, Switzerland)* 9:83. 10.3390/antiox9010083 31963866 PMC7022588

[B57] Li X. Feng K. Li J. Yu D. Fan Q. Tang T. (2017). Curcumin inhibits apoptosis of chondrocytes through activation ERK1/2 signaling pathways induced autophagy. *Nutrients* 9:414. 10.3390/nu9040414 28430129 PMC5409753

[B58] Li Z. Cheng J. Liu J. (2020). Baicalin protects human OA chondrocytes against IL-1β-induced apoptosis and ECM degradation by activating autophagy via MiR-766-3p/AIFM1 axis. *Drug Design Dev. Ther.* 14 2645–2655. 10.2147/DDDT.S255823 32753846 PMC7353997

[B59] Loeser R. F. (2011). Aging and osteoarthritis. *Curr. Opin. Rheumatol.* 23 492–496. 10.1097/BOR.0b013e3283494005 21709557 PMC3377970

[B60] Loeser R. F. Carlson C. S. Del Carlo M. Cole A. (2002). Detection of nitrotyrosine in aging and osteoarthritic cartilage: correlation of oxidative damage with the presence of interleukin-1? and with chondrocyte resistance to insulin-like growth factor 1. *Arthritis Rheum.* 46 2349–2357. 10.1002/art.10496 12355482

[B61] López de Figueroa P. Lotz M. K. Blanco F. J. Caramés B. (2015). Autophagy activation and protection from mitochondrial dysfunction in human chondrocytes. *Arthritis Rheumatol. (Hoboken, N.J.)* 67 966–976. 10.1002/art.39025 25605458 PMC4380780

[B62] Lotz M. K. Caramés B. (2011). Autophagy and cartilage homeostasis mechanisms in joint health, aging and OA. *Nat. Rev. Rheumatol.* 7 579–587. 10.1038/nrrheum.2011.109 21808292 PMC3192496

[B63] Löw P. (2011). The role of ubiquitin-proteasome system in ageing. *Gen. Comp. Endocrinol.* 172 39–43. 10.1016/j.ygcen.2011.02.005 21324320

[B64] Luo P. Gao F. Niu D. Sun X. Song Q. Guo C. (2019). The role of autophagy in chondrocyte metabolism and osteoarthritis: a comprehensive research review. *BioMed Res. Int.* 2019:5171602. 10.1155/2019/5171602 31111057 PMC6487163

[B65] Ma C.-H. Wu C.-H. Jou I.-M. Tu Y.-K. Hung C.-H. Chou W.-C. (2019). PKR promotes oxidative stress and apoptosis of human articular chondrocytes by causing mitochondrial dysfunction through p38 MAPK activation-PKR activation causes apoptosis in human chondrocytes. *Antioxidants (Basel, Switzerland)* 8:370. 10.3390/antiox8090370 31484360 PMC6769915

[B66] Marmotti A. Rossi R. Castoldi F. Roveda E. Michielon G. Peretti G. M. (2015). PRP and articular cartilage: a clinical update. *BioMed Res. Int.* 2015:542502. 10.1155/2015/542502 26075244 PMC4436454

[B67] Maroudas A. Bayliss M. T. Uchitel-Kaushansky N. Schneiderman R. Gilav E. (1998). Aggrecan turnover in human articular cartilage: use of aspartic acid racemization as a marker of molecular age. *Arch. Biochem. Biophys.* 350 61–71. 10.1006/abbi.1997.0492 9466821

[B68] Martin J. A. Buckwalter J. A. (2001). Telomere erosion and senescence in human articular cartilage chondrocytes. *J. Gerontol. Ser. A Biol. Sci. Med. Sci.* 56 B172–B179. 10.1093/gerona/56.4.b172 11283188

[B69] McCarthy H. E. Bara J. J. Brakspear K. Singhrao S. K. Archer C. W. (2012). The comparison of equine articular cartilage progenitor cells and bone marrow-derived stromal cells as potential cell sources for cartilage repair in the horse. *Vet. J.* 192 345–351. 10.1016/j.tvjl.2011.08.036 21968294

[B70] McCulloch K. Litherland G. J. Rai T. S. (2017). Cellular senescence in osteoarthritis pathology. *Aging Cell* 16 210–218. 10.1111/acel.12562 28124466 PMC5334539

[B71] Mollano A. V. Martin J. A. Buckwalter J. A. (2002). Chondrocyte senescence and telomere regulation: implications in cartilage aging and cancer (a brief review). *Iowa Orthop. J.* 22 1–7. 12180600 PMC1888369

[B72] Moussa M. Lajeunesse D. Hilal G. El Atat O. Haykal G. Serhal R. (2017). Platelet rich plasma (PRP) induces chondroprotection via increasing autophagy, anti-inflammatory markers, and decreasing apoptosis in human osteoarthritic cartilage. *Exp. Cell Res.* 352 146–156. 10.1016/j.yexcr.2017.02.012 28202394

[B73] Muñoz-Najar U. Sedivy J. M. (2011). Epigenetic control of aging. *Antioxid. Redox Signal.* 14 241–259. 10.1089/ars.2010.3250 20518699 PMC3014766

[B74] Musumeci G. Szychlinska M. A. Mobasheri A. (2015). Age-related degeneration of articular cartilage in the pathogenesis of osteoarthritis: molecular markers of senescent chondrocytes. *Histol. Histopathol.* 30 1–12. 10.14670/HH-30.1 25010513

[B75] Nicoliche T. Maldonado D. C. Faber J. Da Silva M. C. P. (2020). Evaluation of the articular cartilage in the knees of rats with induced arthritis treated with curcumin. *PLoS One* 15:0230228. 10.1371/journal.pone.0230228 32163510 PMC7067390

[B76] Nogueira-Recalde U. Lorenzo-Gómez I. Blanco F. J. Loza M. I. Grassi D. Shirinsky V. (2019). Fibrates as drugs with senolytic and autophagic activity for osteoarthritis therapy. *EBioMedicine* 45 588–605. 10.1016/j.ebiom.2019.06.049 31285188 PMC6642320

[B77] Ong A. L. C. Ramasamy T. S. (2018). Role of Sirtuin1-p53 regulatory axis in aging, cancer and cellular reprogramming. *Ageing Res. Rev.* 43 64–80. 10.1016/j.arr.2018.02.004 29476819

[B78] Pal B. Endisha H. Zhang Y. Kapoor M. (2015). mTOR: a potential therapeutic target in osteoarthritis? *Drugs R D* 15 27–36. 10.1007/s40268-015-0082-z 25688060 PMC4359178

[B79] Pan Y. Chen D. Lu Q. Liu L. Li X. Li Z. (2017). Baicalin prevents the apoptosis of endplate chondrocytes by inhibiting the oxidative stress induced by H2O2. *Mol. Med. Rep.* 16 2985–2991. 10.3892/mmr.2017.6904 28677799

[B80] Panagiotou N. Wayne Davies R. Selman C. Shiels P. G. (2016). Microvesicles as vehicles for tissue regeneration: changing of the guards. *Curr. Pathobiol. Rep.* 4 181–187. 10.1007/s40139-016-0115-5 27882267 PMC5101251

[B81] Patel S. Dhillon M. S. Aggarwal S. Marwaha N. Jain A. (2013). Treatment with platelet-rich plasma is more effective than placebo for knee osteoarthritis: a prospective, double-blind, randomized trial. *Am. J. Sports Med.* 41 356–364. 10.1177/0363546512471299 23299850

[B82] Qin N. Wei L. Li W. Yang W. Cai L. Qian Z. (2017). Local intra-articular injection of resveratrol delays cartilage degeneration in C57BL/6 mice by inducing autophagy via AMPK/mTOR pathway. *J. Pharmacol. Sci.* 134 166–174. 10.1016/j.jphs.2017.06.002 28669597

[B83] Raeissadat S. A. Rayegani S. M. Babaee M. Ghorbani E. (2013). The effect of platelet-rich plasma on pain, function, and quality of life of patients with knee osteoarthritis. *Pain Res. Treat.* 2013:165967. 10.1155/2013/165967 24386565 PMC3872432

[B84] Ratajczak M. Z. (2011). The emerging role of microvesicles in cellular therapies for organ/tissue regeneration. *Nephrol Dial Transpl.* 26 1453–1456. 10.1093/ndt/gfr165 21531733

[B85] Rim Y. A. Nam Y. Ju J. H. (2020). The role of chondrocyte hypertrophy and senescence in osteoarthritis initiation and progression. *Int. J. Mol. Sci.* 21:2358. 10.3390/ijms21072358 32235300 PMC7177949

[B86] Ritschka B. Storer M. Mas A. Heinzmann F. Ortells M. C. Morton J. P. (2017). The senescence-associated secretory phenotype induces cellular plasticity and tissue regeneration. *Genes Dev.* 31 172–183. 10.1101/gad.290635.116 28143833 PMC5322731

[B87] Ruiz-Romero C. Calamia V. Mateos J. Carreira V. Martínez-Gomariz M. Fernández M. (2009). Mitochondrial dysregulation of osteoarthritic human articular chondrocytes analyzed by proteomics: a decrease in mitochondrial superoxide dismutase points to a redox imbalance. *Mol. Cell. Proteom. MCP* 8 172–189. 10.1074/mcp.M800292-MCP200 18784066 PMC2713027

[B88] Samuel S. Ahmad R. E. Ramasamy T. S. Karunanithi P. Naveen S. V. Kamarul T. (2019). Platelet-rich concentrate in serum-free medium enhances cartilage-specific extracellular matrix synthesis and reduces chondrocyte hypertrophy of human mesenchymal stromal cells encapsulated in alginate. *Platelets* 30 66–74. 10.1080/09537104.2017.1371287 29090639

[B89] Samuel S. Ahmad R. E. Ramasamy T. S. Manan F. Kamarul T. (2018). Platelet rich concentrate enhances mesenchymal stem cells capacity to repair focal cartilage injury in rabbits. *Injury* 49 775–783. 10.1016/j.injury.2018.02.020 29503013

[B90] Saris T. F. F. de Windt T. S. Kester E. C. Vonk L. A. Custers R. J. H. Saris D. B. F. (2021). Five-year outcome of 1-stage cell-based cartilage repair using recycled autologous chondrons and allogenic mesenchymal stromal cells: a first-in-human clinical trial. *Am. J. Sports Med.* 49 941–947. 10.1177/0363546520988069 33591794

[B91] Savvides S. N. Scheiwein M. Böhme C. C. Arteel G. E. Karplus P. A. Becker K. (2001). Crystal structure of the antioxidant enzyme glutathione reductase inactivated by peroxynitrite. *J. Biol. Chem.* 277 2779–2784. 10.1074/jbc.m108190200 11705998

[B92] Schulze-Tanzil G. De Souza P. Villegas Castrejon H. John T. Merker H. J. Scheid A. (2002). Redifferentiation of dedifferentiated human chondrocytes in high-density cultures. *Cell Tissue Res.* 308 371–379. 10.1007/s00441-002-0562-7 12107430

[B93] Serrano R. L. Chen L. Y. Lotz M. K. Liu-Bryan R. Terkeltaub R. (2018). Impaired proteasomal function in human osteoarthritic chondrocytes can contribute to decreased levels of SOX9 and Aggrecan. *Arthritis Rheumatol.* 70 1030–1041. 10.1002/art.40456 29457374 PMC6019618

[B94] Shao J. H. Fu Q. W. Li L. X. Zhou R. Liu N. Peng J. H. (2020). Prx II reduces oxidative stress and cell senescence in chondrocytes by activating the p16-CDK4/6-pRb-E2F signaling pathway. *Eur. Rev. Med. Pharmacol. Sci.* 24 3448–3458. 10.26355/eurrev_202004_20802 32329817

[B95] Shen C. Cai G.-Q. Peng J.-P. Chen X.-D. (2015). Autophagy protects chondrocytes from glucocorticoids-induced apoptosis via ROS/Akt/FOXO3 signaling. *Osteoarthritis Cartilage* 23 2279–2287. 10.1016/j.joca.2015.06.020 26165503

[B96] Tamayo M. Mosquera A. Rego I. Blanco F. J. Gosálvez J. Fernández J. L. (2011). Decreased length of telomeric DNA sequences and increased numerical chromosome aberrations in human osteoarthritic chondrocytes. *Mutat. Res. Fundam. Mol. Mech. Mutagen.* 708 50–58. 10.1016/j.mrfmmm.2011.01.007 21291897

[B97] Tan L. Register T. C. Yammani R. R. (2020). Age-related decline in expression of molecular chaperones induces endoplasmic reticulum stress and chondrocyte apoptosis in articular cartilage. *Aging Dis.* 11 1091–1102. 10.14336/AD.2019.1130 33014525 PMC7505268

[B98] Tetta C. Ghigo E. Silengo L. Deregibus M. C. Camussi G. (2013). Extracellular vesicles as an emerging mechanism of cell-to-cell communication. *Endocrine* 44 11–19. 10.1007/s12020-012-9839-0 23203002 PMC3726927

[B99] Turinetto V. Vitale E. Giachino C. (2016). Senescence in human mesenchymal stem cells: functional changes and implications in stem cell-based therapy. *Int. J. Mol. Sci.* 17:1164. 10.3390/ijms17071164 27447618 PMC4964536

[B100] Ukai T. Sato M. Akutsu H. Umezawa A. Mochida J. (2012). MicroRNA-199a-3p, microRNA-193b, and microRNA-320c are correlated to aging and regulate human cartilage metabolism. *J. Orthop. Res.* 30 1915–1922. 10.1002/jor.22157 22674437

[B101] van Deursen J. M. (2014). The role of senescent cells in ageing. *Nature* 509 439–446. 10.1038/nature13193 24848057 PMC4214092

[B102] Varela-Eirín M. Carpintero-Fernández P. Sánchez-Temprano A. Varela-Vázquez A. Paíno C. L. Casado-Díaz A. (2020). Senolytic activity of small molecular polyphenols from olive restores chondrocyte redifferentiation and promotes a pro-regenerative environment in osteoarthritis. *Aging* 12 15882–15905. 10.18632/aging.103801 32745074 PMC7485729

[B103] Vellai T. Takács-Vellai K. Sass M. Klionsky D. J. (2009). The regulation of aging: does autophagy underlie longevity? *Trends Cell Biol.* 19 487–494. 10.1016/j.tcb.2009.07.007 19726187 PMC2755611

[B104] Verzijl N. DeGroot J. Thorpe S. R. Bank R. A. Shaw J. N. Lyons T. J. (2000). Effect of collagen turnover on the accumulation of advanced glycation end products. *J. Biol. Chem.* 275 39027–39031. 10.1074/jbc.m006700200 10976109

[B105] Vinatier C. Domínguez E. Guicheux J. Caramés B. (2018). Role of the inflammation-autophagy-senescence integrative network in osteoarthritis. *Front. Physiol.* 9:706. 10.3389/fphys.2018.00706 29988615 PMC6026810

[B106] Wang F.-S. Kuo C.-W. Ko J.-Y. Chen Y.-S. Wang S.-Y. Ke H.-J. (2020). Irisin mitigates oxidative stress, chondrocyte dysfunction and osteoarthritis development through regulating mitochondrial integrity and autophagy. *Antioxidants (Basel, Switzerland)* 9:810. 10.3390/antiox9090810 32882839 PMC7555738

[B107] Wang Y. Yu D. Liu Z. Zhou F. Dai J. Wu B. (2017). Exosomes from embryonic mesenchymal stem cells alleviate osteoarthritis through balancing synthesis and degradation of cartilage extracellular matrix. *Stem Cell Res. Ther.* 8:189. 10.1186/s13287-017-0632-0 28807034 PMC5556343

[B108] Wang Y. Zhao X. Lotz M. Terkeltaub R. Liu-Bryan R. (2015). Mitochondrial biogenesis is impaired in osteoarthritis chondrocytes but reversible via peroxisome proliferator-activated receptor γ coactivator 1α. *Arthritis Rheumatol. (Hoboken, N.J.)* 67 2141–2153. 10.1002/art.39182 25940958 PMC4519411

[B109] Wei F. Y. Lee J. K. Wei L. Qu F. Zhang J. Z. (2017). Correlation of insulin-like growth factor 1 and osteoarthritic cartilage degradation: a spontaneous osteoarthritis in guinea-pig. *Eur. Rev. Med. Pharmacol. Sci.* 21 4493–4500. 29131268 PMC6100760

[B110] Williams R. Khan I. M. Richardson K. Nelson L. McCarthy H. E. Analbelsi T. (2010). Identification and clonal characterisation of a progenitor cell sub-population in normal human articular cartilage. *PLoS One* 5:e13246. 10.1371/journal.pone.0013246 20976230 PMC2954799

[B111] Wilson B. Novakofski K. D. Donocoff R. S. Liang Y.-X. A. Fortier L. A. (2014). Telomerase activity in articular chondrocytes is lost after puberty. *Cartilage* 5 215–220. 10.1177/1947603514537518 26069700 PMC4335769

[B112] Xie J. Lin J. Wei M. Teng Y. He Q. Yang G. (2019). Sustained Akt signaling in articular chondrocytes causes osteoarthritis via oxidative stress-induced senescence in mice. *Bone Res.* 7:23. 31646013 10.1038/s41413-019-0062-y PMC6804644

[B113] Xu M. Pirtskhalava T. Farr J. N. Weigand B. M. Palmer A. K. Weivoda M. M. (2018). Senolytics improve physical function and increase lifespan in old age. *Nat. Med.* 24 1246–1256. 10.1038/s41591-018-0092-9 29988130 PMC6082705

[B114] Yang H. Chen C. Chen H. Duan X. Li J. Zhou Y. (2020). Navitoclax (ABT263) reduces inflammation and promotes chondrogenic phenotype by clearing senescent osteoarthritic chondrocytes in osteoarthritis. *Aging* 12 12750–12770. 10.18632/aging.103177 32611834 PMC7377880

[B115] Yun M. H. Davaapil H. Brockes J. P. (2015). Recurrent turnover of senescent cells during regeneration of a complex structure. *ELife* 4:e05505. 10.7554/eLife.05505 25942455 PMC4434796

[B116] Zhang M. Wang J. (2015). Epigenetics and osteoarthritis. *Genes Dis.* 2 69–75. 10.1016/j.gendis.2014.12.005 25961070 PMC4421878

[B117] Zhang S. Teo K. Y. W. Chuah S. J. Lai R. C. Lim S. K. Toh W. S. (2019). MSC exosomes alleviate temporomandibular joint osteoarthritis by attenuating inflammation and restoring matrix homeostasis. *Biomaterials* 200 35–47. 10.1016/j.biomaterials.2019.02.006 30771585

[B118] Zhang Y. Morgan B. J. Smith R. Fellows C. R. Thornton C. Snow M. (2017). Platelet-rich plasma induces post-natal maturation of immature articular cartilage and correlates with LOXL1 activation. *Sci. Rep.* 7:3699. 10.1038/s41598-017-02297-9 28623328 PMC5473810

[B119] Zhou H. W. (2004). Recovery of function in osteoarthritic chondrocytes induced by p16INK4a-specific siRNA in vitro. *Rheumatology* 43 555–568. 10.1093/rheumatology/keh127 15026580

[B120] Zhuo Q. Yang W. Chen J. Wang Y. (2012). Metabolic syndrome meets osteoarthritis. *Nat. Rev. Rheumatol.* 8 729–737. 10.1038/nrrheum.2012.135 22907293

